# From infrastructure to impact: why foundations matter in digital health

**DOI:** 10.2471/BLT.24.293085

**Published:** 2025-02-01

**Authors:** Alain Labrique

**Affiliations:** a Department of Digital Health and Innovation, World Health Organization, Avenue Appia 20, 1211 Geneva 27, Switzerland.

Global health crises, such as the coronavirus disease 2019 (COVID-19) pandemic, antimicrobial resistance and the effects of climate change have underscored the transformative potential of digital health technologies as well as the negative consequences of underinvestment in foundational digital public infrastructure, such as digital identity, payment systems and data exchanges.^1^ Such infrastructure constitutes necessary building blocks for digital health systems. Two decades of digital health pilot projects have bolstered confidence that digital investments could be as transformative to health as they have been in other sectors – from banking to transport. Over 120 countries have a national digital health strategy or action plan.^2^ However, achieving digital transformation through robust, interconnected and equitable digital health systems requires political will, appropriate local governance and considerable, sustained investment.

Investments in digital public infrastructure complement national health priorities, often determining whether health systems can respond effectively to crises and deliver equitable care.^3^ The COVID-19 pandemic exposed critical gaps, revealing that even the best technologies fall short in agility and responsiveness to public health shocks when local decision-making, technical capacity and public trust are inadequate.^4^ Local capacity to adapt systems must exist, as should the policy architecture that enables people to entrust their health information and quality of care to digital systems. Recent studies have shown that technology use and adoption increases with perceived improvements in personal empowerment or agency and with evidence of efficacy.^5^

During the COVID-19 pandemic, countries with established digital health systems supported by strong policies and governance adapted swiftly – tracking infections, enabling remote consultations and supporting vaccination roll-outs. However, countries with fragmented systems, weakened by non-interoperable platforms and fragile data governance, struggled. Theirs was not a failure of technology but of policy, infrastructure and investment. Systemic digital transformation, as seen in other sectors, happens when countries invest in digital foundations that foster real-time data sharing, coordinated service delivery and patient-centred care.

The World Health Organization (WHO) has been promoting digital health for over two decades, serving as the Secretariat for the Member State *Global strategy on digital health (2020–2025)*. In 2023, WHO launched the Global Initiative on Digital Health,^6^ a platform designed to transition digital health strategies into impactful systems. This initiative emphasizes political leadership, public infrastructure and sustainable funding to help countries build accountable, data-driven digital health systems.

However, for the strategy to succeed, strong legal frameworks, dedicated agencies and consistent investment are needed. India’s Ayushman Bharat Digital Mission^7^ and Kenya’s Digital Health Bill^8^ exemplify such leadership by embedding digital health governance into national policy. Brazil, Estonia, Indonesia, Malaysia and Rwanda have developed strong digital foundations^2^ that simultaneously invest in public trust and public infrastructure to present the benefits of digital health for both developers and users. These government-led initiatives often took years, if not decades, to plan and systematically implement, outlasting multiple political cycles.

Furthermore, digital transformation goes beyond digitizing outdated processes; it is also about reimagining how care is delivered. Saudi Arabia’s Seha Virtual Hospital^9^ connects patients with specialists through telemedicine, highlighting how digital models can expand care access in even the most remote areas, prioritizing patient satisfaction and quality-of-care outcomes.

Despite progress, financing for digital health remains insufficient. The World Bank has invested nearly 4 billion United States dollars (US$) in digital health over the past decade, but analyses suggest that US$ 12.5 billion more are needed over the next five years to support 78 low- and middle-income countries.^10^ Funding must enable countries to develop robust digital and data governance, and launch and maintain foundational and health-specific digital health public infrastructure, while ensuring universal connectivity and digital inclusion.^11^

Digital health transformation is a complex, long-term process requiring political resolve, dedicated investment and an unwavering commitment to equity. As countries shift from strategy to implementation, WHO and its partners remain committed to supporting this journey.
